# A transmission relationship investigation of HIV infection through male-to-male sex among a case of left-behind children with heterosexual orientation in Zhejiang Province of China

**DOI:** 10.3389/fpubh.2026.1619949

**Published:** 2026-01-16

**Authors:** Zhigang Hou, Jiafeng Zhang, Hao Feng, Zhongwen Chen, Qin Fan, Xiaohong Pan, Guoying Zhu, Rui Ge

**Affiliations:** 1 Jiaxing Center for Disease Control and Prevention, Jiaxing, China; 2 Department of HIV/AIDS Control and Prevention, Zhejiang Provincial Center for Disease Control and Prevention, Hangzhou, Zhejiang, China; 3 Zhejiang AIDS/STD Prevention Association, Hangzhou, Zhejiang, China

**Keywords:** HIV, left-behind children, MSM, phylogenetic analysis, transmission relationship

## Abstract

**Objective:**

This study seeks to trace the source of HIV infection in a male adolescent (P1) with a newly recognized heterosexual orientation in Zhejiang Province on August 23, 2019.

**Methods:**

An in-depth epidemiological interview was conducted with P1 and all sexual partners. For HIV-infected partners, sequential partner tracing was performed until no new HIV cases were detected. The survey assessed high-risk sexual behavior, HIV testing and treatment history, and demographic details. Serum samples were collected for HIV antibody testing, and plasma samples from HIV-positive individuals were taken before antiviral therapy. HIV RNA was extracted, and specific gene regions were amplified, cloned, and sequenced. Phylogenetic trees were constructed using MEGA V6.0 to identify HIV subtypes, calculate genetic distances, and analyze genetic associations. Genetic similarity was calculated using BioEdit V7.2.0.

**Results:**

The results indicated that P1, a 16-year-old male dropout and left-behind child, first tested positive for HIV-1 antibodies on August 23, 2019. P1’s heterosexual partner, W1, is HIV-negative. P1 had a same-sex partner, P2, who tested positive on November 9, 2018. P2 is an adult, did not communicate with P1 about his HIV status and had unprotected sex with P1 before P1 tested HIV positive. P2 reported seven male sexual partners in the same district, P1 was one of them and all other sexual partners were HIV-negative. Among these seven partners, two were with divorced parents, six were dropouts, and three were left-behind children. Phylogenetic analysis revealed that both P1 and P2 had CRF01_AE/CRF55_01B/CRF07_BC recombinant HIV. The genetic similarity of the P1 sample and the two P2 samples in the gag, pol, and env gene regions was 98.8%, 99.5%, and 98.8%, and 99.0%, 99.7%, and 99.0%, respectively. The genetic similarity between two samples of P2 in these regions was 98.7, 99.6, and 99.9%. In the gag and env gene regions, interwoven branching patterns were observed, which are indicative of shared transmission chains. For the pol gene region, the C1 paraphyletic sequences of P1 and P2 clustered within the same evolutionary branch, with a bootstrap value of 100% and a mean genetic distance of 0.003 within this cluster.

**Conclusion:**

Epidemiological and phylogenetic analyses suggest that P1 may have contracted HIV through unprotected sex with an HIV-positive P2 who did not disclose his HIV infection.

## Introduction

Men who have sex with men (MSM) constitute a priority demographic for both HIV infection and transmission (1). A comprehensive search showed that HIV prevalence in MSM ranged from 3.0% in the Middle East and North Africa region to 25.4% in the Caribbean, and HIV infection levels in MSM were substantially higher than those in non-MSM individuals (2). In China, the HIV prevalence among MSM increased from 4.9% in 2008 to 7.9% in 2020 (3). Notably, in 2022, over 60% of the newly diagnosed HIV infected from the developed cities in China were due to male-to-male transmission (4, 5). In China, the MSM population is estimated to exceed 20 million individuals (6). A meta-analysis found that the national HIV incidence among Chinese MSM was 5.0 per 100 person-years (95% CI: 4.1–5.8%) (7), which was much higher than that of any other population in China. A fast-spreading HIV epidemic among MSM constitutes a challenge to efforts to control the HIV pandemic.

Previous studies have shown that MSM people mainly have sex with same-sex sexually oriented men, but they also have sex with heterosexual sexually oriented men through money transactions, violence and coercion (6, 8). Underage heterosexually oriented men, especially left-behind children and out-of-school children, are at risk of being induced or coerced by money to engage in same-sex sexual behavior due to insufficient supervision and education, which in turn elevates the risk of HIV infection and transmission (8, 9). Left-behind children refer to minors under the age of 16 who are unable to live normally with their parents because both parents go out to work for a long time, or one parent goes out while the other is incapable of supervision. In 2015, there were 68.77 million left-behind children in China, including 40.51 million in rural areas (10, 11). Epidemiological characteristics of HIV transmission between MSM and heterosexual men have been studied in China (12, 13). However, there are no case reports on the relationship between MSM and heterosexual men who left behind children regarding HIV transmission. Left-behind children’s long-term lack of parental emotional support and social guidance has led to a decrease in their social trust, but at the same time, they are also more likely to rely on external relationships to fill the emotional gap, increasing the risk of being exploited. A little kindness or monetary temptation from strangers can easily subject left-behind children to deception, while the poor living conditions and economic status of most left-behind children further increase the risk of these left-behind children being victimized by adult MSM through monetary enticement or violent coercion. In recent years, the application of phylogenetic analysis techniques has provided new support for determining the transmission relationship between HIV-infected individuals through molecular biology (14).

In a phylogenetic tree, a cluster represents a group of sequences from potential transmission partners. Thus, the genetic relatedness of HIV-1 can reflect the relationships between infected individuals, based on which the potential genetic transmission networks can be inferred (15, 16). Recently, with advances in molecular epidemiology, analyses of genetic transmission networks can help HIV researchers and public health professionals understand how HIV is spread within and between populations and further deliver efficient and effective interventions (17–19). Thus, genetic transmission network analyses are useful for revealing HIV acquisition risk factors for MSM at the network level. Since 2017, surveillance efforts in Zhejiang Province have identified the formation of a molecular transmission network of HIV among MSM populations, as well as among both homosexual and heterosexually oriented males, including underage individuals (12, 13, 20).

This research examines a case involving a minor male infected with HIV who filed a lawsuit against an adult male, also HIV-positive, for the alleged intentional transmission of the virus in August 2019 in Zhejiang Province. The case involved several left-behind children (LBC). Utilizing a combination of epidemiological investigation and evolutionary analysis of HIV molecular sequences, this study traces the infection pathways among the underage individuals affected. Furthermore, it analyzes the characteristics of the associated sexual transmission network and offers recommendations for effective intervention strategies.

## Materials and methods

### Case information and samples

P1 (Complainant) was diagnosed as HIV-1 positive on August 23, 2019, when he was 16 years old. P2 (Respondent) was diagnosed as HIV-1 positive on November 16, 2018, when he was 53 years old, by a municipal HIV testing facility in Zhejiang Province (Jiaxing City Center for Disease Control and Prevention, JXCDC). P1 alleged that P2 had unlawfully infected him with HIV-1 via unprotected sexual contact without disclosing his HIV-positive status. Thereafter, a retrospective epidemiological investigation was carried out by the corresponding CDCs responsible for case follow-up. CDC staff administered a questionnaire to obtain demographic and risk behavior information after an HIV diagnosis. The questionnaire included demographic characteristics (age, gender, current residence, registered residence, migration), risk behavior characteristics (location, time range, condom use), characteristics of sexual partners (gender, number, methods of seeking sexual partners), and HIV testing history. Medical records related to HIV testing (e.g., HIV testing during invasive examination) were also collected.

Plasma samples before antiretroviral therapy of P1 and P2 were collected and isolated on August 27, 2019 and September 1, 2019, respectively, and the samples were referred to as P1 and P2-1. Plasma samples first retained after P2 was confirmed HIV-1 antibody-positive on November 16, 2018, and the samples were referred to as P2-2, which were retrieved from the laboratory of the Zhejiang Center for Disease Control and Prevention. Three plasma samples were analyzed by the professional staff of the Zhejiang Center for Disease Control and Prevention. Plasma samples were subjected to nucleic acid amplification, molecular clonal sequencing and phylogenetic analysis. The P1 and P2 plasma samples collected within 1 month after diagnosis were available in the HIV-positive sample bank of Zhejiang Provincial Center for Disease Control and Prevention (ZJCDC).

### Epidemiological survey

One-on-one on-site interviews and surveys were conducted by occupational physicians who had been engaged in HIV prevention and control work for more than 3 years at the CDC in Jiaxing City, Zhejiang Province, with the survey respondents. The content of the interview included the sex, age, household registration, guardianship, family structure and other socio-demographic characteristics of the respondents; testing history such as HIV test time, test location, test results; antiretroviral treatment history such as HIV post-exposure prophylaxis or post-diagnostic treatment; and history of sexual behavior such as previous sexual activity, time of occurrence, place of occurrence, and whether or not condoms were used.

### Amplification of HIV-1 gene fragments

Viral RNA was extracted from 140 μL of plasma using the QIAamp Viral RNA Mini Kit (Qiagen, Valencia, CA, United States) according to the manufacturer’s instructions. RNA samples were directly subjected to nested polymerase chain reactions (PCR) to generate fragments of gag (HXB2: 781 → 1861; encoding portions of p17 and p24), pol (HXB2: 2147 → 3462; encoding the protease and the first 299 residues of reverse transcriptase) and env (HXB2: 7002 → 7541, encoding the V3-V4 region). The details of amplification and sequencing were as previously described (21). At least 20 clones per partial HIV-1 gene region were sequenced for each subject at each time point. Positive and negative controls were established through nucleic acid extraction, PCR amplification and molecular cloning. No nucleic acid cross-contamination occurred during the experiment.

### Phylogenetic analysis

The assembly of the different sequences generated from the same gene region of each sample was performed using the DNA sequence analysis software Sequencher v5.4.6 (Gene Codes, Ann Arbor, MI). ClustalW multiple alignments were performed using Bio-Edit v7.0 software. Reference sequences were obtained from the Los Alamos National Laboratory (LANL) database,^1^ which covers the major HIV-1 subtypes/circulating recombinant forms (CRFs). Genetic distances between nucleic acid sequences were calculated using MEGA v6.0 (22), and nucleic acid sequences of the same subtype strain with the highest molecularly monitored sequence homology among newly reported HIV-positive infections in Jiaxing City in 2018 were selected as controls. The phylogenetic tree was constructed, and the bootstrap value of the evolutionary tree was set to 90%, and the genetic distance between sequences in the cluster was thresholded at 0.015. When the bootstrap value was ≥90%, the average genetic distance within molecular clusters ≤0.015 was defined as the existence of transmission association between gene sequences, and the degree of transmission association was analyzed in conjunction with the phenomenon of concatenation to determine the direction of transmission.

### Statistical analysis

Statistical analyses were conducted with SPSS v22.0 software (IBM, Armonk, NY). A *p* value < 0.05 was considered statistically significant.

## Results

### Epidemiological information and serological testing

**Figure 1:**
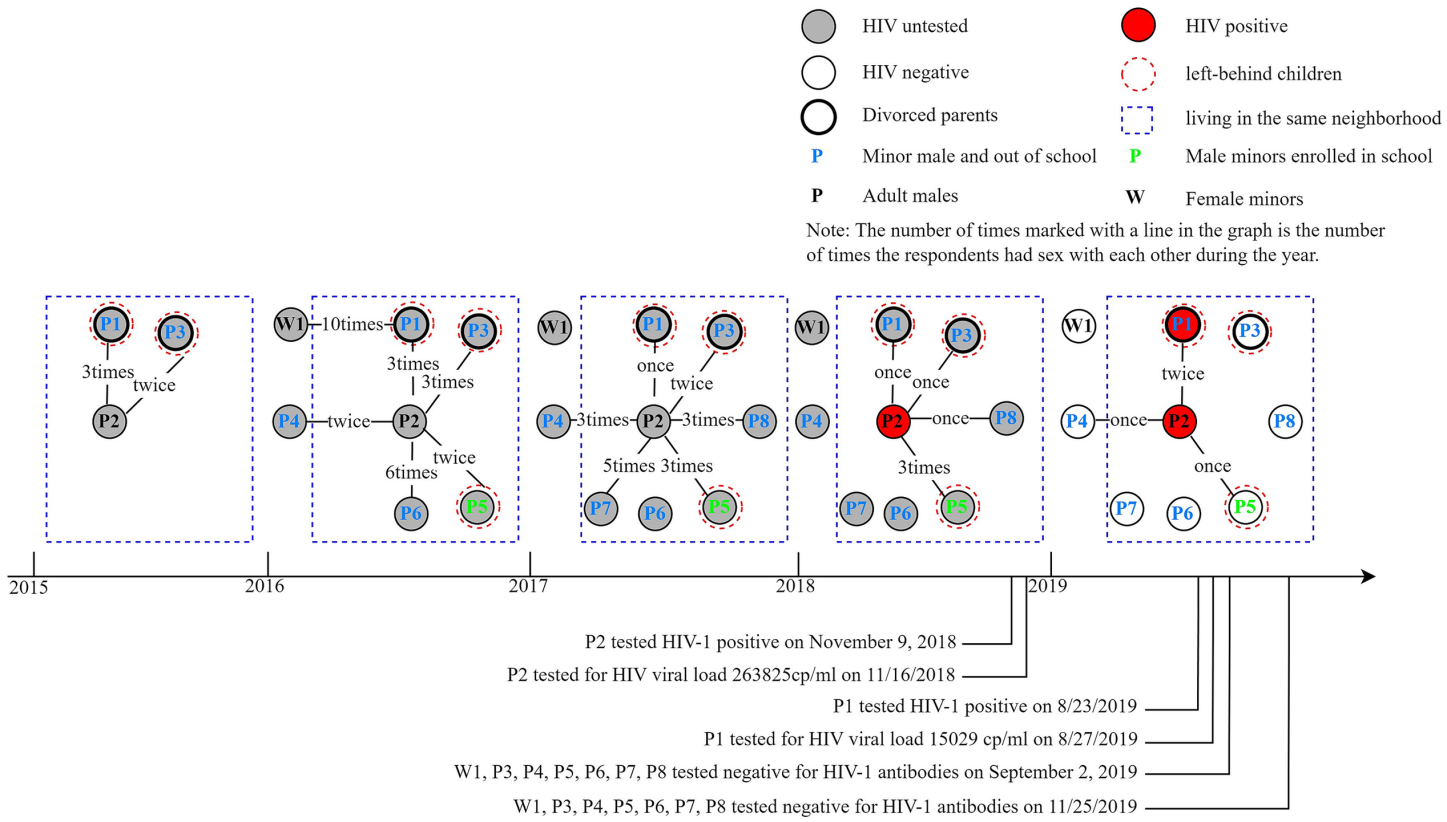
Relationships and general sociological characteristics of sexual behavior among survey respondents.

Epidemiological Profile of Subject P1: Subject P1 is a 16-year-old male who has discontinued formal education. His parents are divorced; his mother has remarried, and his father is currently incarcerated. P1 resides with his grandparents in a township in Jiaxing City and identifies as heterosexual. P1 had no documented history of HIV positivity and was first identified as HIV-1 antibody-positive during a routine health examination on August 23, 2019. P1 reported having had one heterosexual partner (W1) and one homosexual partner, a sexual partner with man to man sexual behavior (P2), as illustrated in Figure 1. According to P1, he engaged in heterosexual intercourse with W1 on ten occasions between March and November 2016, consistently using condoms, and the relationship ended in 2017. P1 reported meeting P2 via the QQ dating platform in April 2015. Between August 2015 and February 2019, P2 engaged in sexual activity with P1, providing monetary compensation ranging from RMB 100 to 400. P1 also reported experiencing hemorrhoids and anal bleeding despite using lubricant during intercourse with P2. P1 did not report any additional male sexual partners. The information provided by P1 regarding his sexual partners aligns with findings from local law enforcement investigations and evidence collection.

Epidemiological Profile of P2, the HIV-Positive Sexual Partner of P1: P2 is a 53-year-old divorced male who relocated from Sichuan Province to a township in Jiaxing City, Zhejiang Province, in April 2015 to work as a temporary laborer at a construction site. This site is located approximately 350 meters in a straight line from the neighborhood where P1 resides. P2 had no prior history of HIV testing until his initial preoperative screening on November 9, 2018, which returned a positive result for the HIV-1 antibody. P2 reported having approximately 20 male sexual partners from various provinces before his arrival in Jiaxing City, although the HIV status of these partners remains unknown. Additionally, P2 had a heterosexual partner, his former spouse, who is HIV-negative and resides in a different province. Upon his arrival in Jiaxing City in 2015, P2 engaged in sexual activities with seven underage male partners (identified as P1, P3, P4, P5, P6, P7, and P8, respectively), as depicted in Figure 1. P2 and P4 became acquainted through the QQ dating platform, while the remaining five partners resided in the same neighborhood as P1 and were introduced to P2 by P1. At the time of their initial sexual encounters with P2, P1 and the other seven individuals were all under the age of 16. Among these individuals, six had discontinued their education, except for P5, who was enrolled in junior high school. P1, P3, and P5 were identified as left-behind children (LBC), with P1 and P3 originating from divorced families. Following a confirmed diagnosis of HIV-1, P2 did not pursue antiretroviral therapy and engaged in unprotected same-sex intercourse with P1 on two occasions, as well as with P4 and P5 once each, without the use of condoms, as illustrated in Figure 1. P2 consistently utilized a substantial amount of lubricant during sexual activities, occasionally used a condom, and recorded videos using a cell phone. Furthermore, all participants indicated that they had never received HIV post-exposure prophylaxis.

On August 23, 2019, P1 tested positive for HIV-1 antibodies, with a CD4 count of 681 cells/μl on August 27 and a viral load of 15,029 copies/ml. P2 tested positive for HIV-1 antibodies on November 9, 2018, with a CD4 count of 242 cells/μl on November 16 and a viral load of 263,824 copies/ml, remaining untreated. A subsequent test on September 1, 2019, revealed a CD4 count of 164 cells/μl and a viral load of 295,743 copies/ml for P2. W1, P3, P4, P5, P6, P7, and P8 tested negative for HIV antibodies on September 2 and November 25.

### Transmission relationship

**Figure 2:**
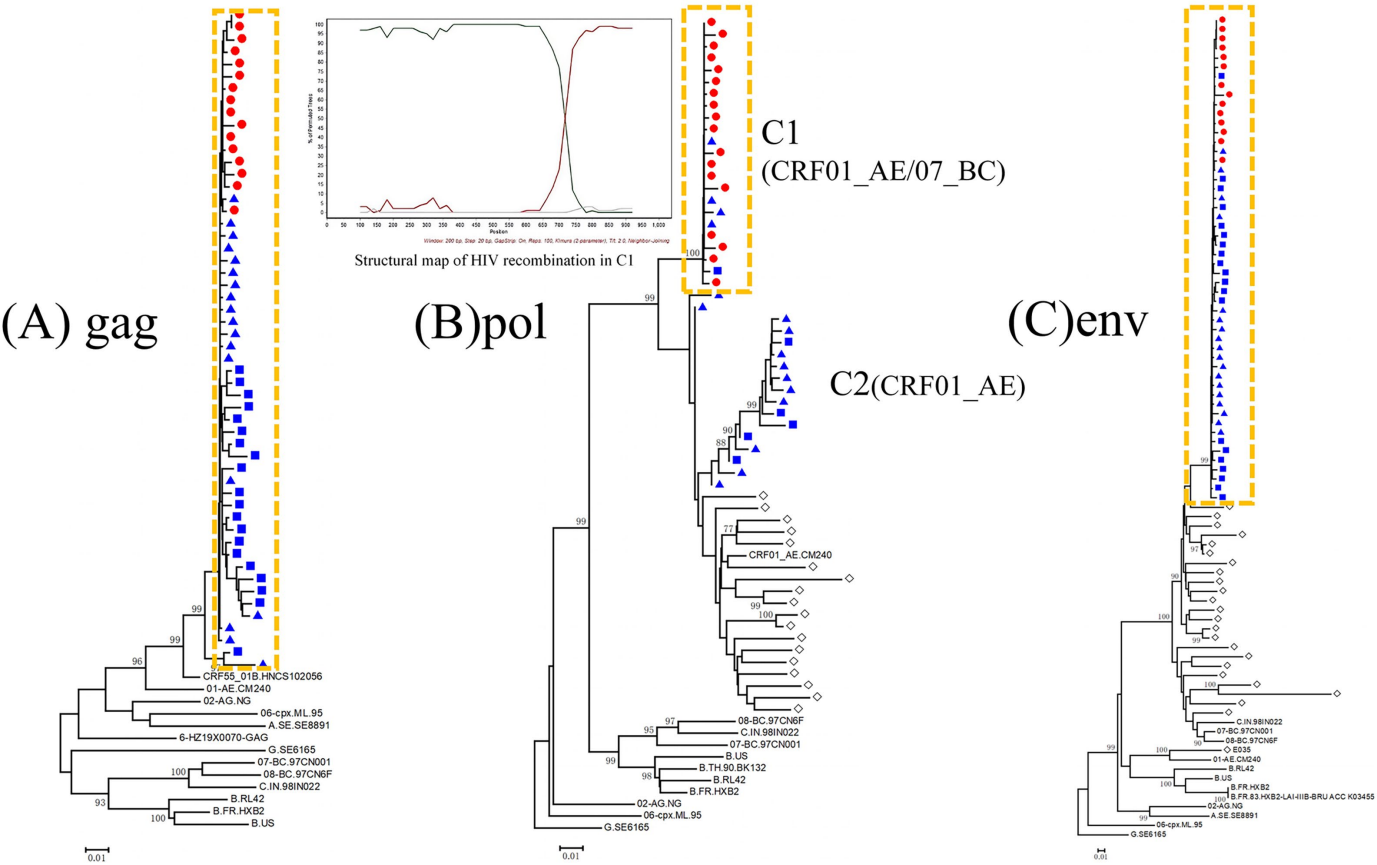
Bayesian phylogenetic tree of consensus sequences from the P1, P2, and local controls. **(A)** Bayesian phylogenetic tree for consensus *gag* sequences and reference sequences. The scale bar indicates 1% nucleotide sequence divergence. **(B)** Bayesian phylogenetic tree for consensus *pol* sequences and relative reference sequences. The scale bar indicates 1% nucleotide sequence divergence. **(C)** Bayesian phylogenetic tree for consensus *env* sequences and relative reference sequences. The scale bar indicates 1% nucleotide sequence divergence. Values on the branches represent the percentage of 1,000 bootstrap replicates and bootstrap values over 70% are shown in the tree. Red round: the sequence from the P1; Blue square: the sequences from the P2-1; Blue triangle: the sequences from the P2-2; Yellow dashed line: HIV gene sequences of the study subjects cluster together in the phylogenetic tree; Rhombic box: locally prevalent strain.

The subtypes of Pl in the gag, pol and env gene regions are CRF55_01B, CRF0l_AE/CRF07_BC and CRF07_BC, respectively. P2-1 and P2-2 have the same subtypes as P1 in both the gag and env gene regions, but there are two mixed subtypes in the pol gene region. One is the CRF01_AE/CRF07_BC recombinant isoform identical to P1, shown as Cl in Figure 2, and the second is the CRF0l_AE isoform, shown as C2 in Figure 2. Both Pl and P2 have the isoform-consistent CRF0l _AE/CRF55_01B/ CRF07_BC recombinant HIV nucleic acid. The recombinant HIV structure of C1 is detailed in Figure 2 (Supplementary Figure S1 for a high-resolution version).

The genetic similarity of P1 and P2-1 in the gag, pol, and env gene regions were 98.8%, 99.5%, and 98.8%, respectively; the genetic similarity of P1 and P2-2 in the gag, pol, and env gene regions were 99.0%, 99.7%, and 99.0%, respectively. The genetic similarity of P2-1 and P2-2 in the gag, pol, and env gene regions were 98.7%, 99.6%, and 99.9%, respectively (Table 1).

**Table 1:** Genomic genetic similarity of quasispecies sequences in the *gag*, *pol*, and *env* gene regions of the commissioned study samples.

Sample ID	Genomic region		
	gag	pol^a^	env
P1 VS P2-1	98.80%	99.50%	98.80%
P1 VS P2-2	99.00%	99.70%	99.00%
P2-1 VS P2-2	98.70%	99.60%	99.90%

a Genetic similarity of C1 quasispecies sequences was calculated for subtype concordance.

Control specimens were selected from a total of 18 nucleic acid sequence specimens of the same subtype strain with the highest molecular monitoring sequence homology among newly reported HIV-positive infected persons in Jiaxing City in 2018. Eighteen control persons were male, same-sex transmission, with an average age (46.39 ± 14.61) years old, and the sampling time was between May–December 2018. The mean within-group genetic distances of P1 and P2 in the pol and env gene regions were 0.003 ± 0.001 and 0.010 ± 0.001, respectively, while the mean within-group genetic distances between them and the control sequences were 0.058 ± 0.008 and 0.130 ± 0.010, respectively (Table 2), with statistically significant differences between the two groups (*p* < 0.001).

**Table 2:** Genetic distances within groups P1 and P2 and between groups with control sequences.

Genomic region	Mean genetic distance within group^a^	Mean genetic distances within groups of sequences with control^a^	*P* ^b^
gag	0.014 ± 0.001	/	/
pol	0.003 ± 0.001	0.058 ± 0.008	*P* < 0.001
env	0.010 ± 0.001	0.130 ± 0.001	*P* < 0.001

a Mean ± SD.

b Rank-sum test.

Epidemiological and phylogenetic analyses highly suggest that P1 acquired HIV through unprotected sexual contact with HIV-positive P2, who had not disclosed his HIV status. In the gag and env gene regions, the paraphyletic sequences of Pl and P2 were in the same evolutionary branch, bootstrap value = 99%, and the average genetic distance within clusters 0.015, and both of them were in paraphyletic phenomenon (Figures 2A,B). In the gag and env gene regions, interwoven branching patterns were observed, which are indicative of shared transmission chains. For the pol gene region, the C1 paraphyletic sequences of P1 and P2 clustered within the same evolutionary branch, with a bootstrap value of 100% and a mean genetic distance of 0.003 within this cluster. Additionally, a sequence concatenation phenomenon was identified in this region.

## Discussion

This is an incident of HIV infection in an underage heterosexual sexually oriented male through men who have sex with men. Based on the epidemiologic investigation and laboratory testing of P1 and his sexual partners and mother, the possibility of P1 contracting HIV through mother-to-child transmission and heterosexual sexual transmission can be excluded. In addition, based on P1’s past medical and behavioral history, P1 was not found to be infected with HIV through blood transfusions, dental extractions, tattoos, etc. P1 self-reported that he had unprotected male-to-male sex only with P2 and two of the incidents occurred after P2 was confirmed to be HIV-positive. HIV subtyping revealed that both Pl and P2 had subtypically concordant recombinant HIV nucleic acid. The genetic similarity between the two specimens of P1 and P2 in the gag, pol, and env gene regions was more than 98.8%, and the higher similarity of the genes represented the higher genetic homology (23, 24). The mean genetic distance within the groups of P1 and P2 in the pol and env gene regions was smaller than the average genetic distance between the groups of P1 and P2 with the control sequences, and the difference was statistically significant (*p* < 0.001). And the smaller genetic distance proves the closer genetic evolutionary relationship between the two (23, 24). The quasispecies sequences of P1 and P2 clustered within the same evolutionary clade across the gag, pol, and env gene regions, with evidence of concatenation events and interwoven branching patterns—hallmarks of shared transmission chains. These findings indicate that the HIV strains infecting P1 and P2 exhibit high genetic homology and evolutionary relatedness, and support a direction of transmission from P2 to P1 (23). The results of epidemiologic investigation and laboratory tests suggest that P1’s HIV infection originated from P2, while P3-P8 were not infected with HIV by P2, partly because they used large doses of “lubricating fluid” during sexual intercourse with P2 and therefore did not have anal or rectal bleeding, while only P1 suffered from hemorrhoids and repeatedly had anal bleeding (23). On the other hand, P2 had sex with P3-P8 mainly before 2018, and P2 did not have sex with P3, P6, P7, and P8 but only had sex with P4 and P5 once each after being confirmed HIV-positive, and had sex with P1 twice, so the risk of infection for P3-P8 was lower than that for P1. In addition, P2 partially used condoms during sex with P3-P8, which also provided some protection against HIV transmission.

Two of the seven minor sexual partners of P2 had divorced parents, and three were LBC under the physical custody of their grandparents. This suggests that unsound family structure and insufficient supervision and care are important factors influencing the emergence of psychological and behavioral problems of minors, especially LBC (25, 26). The community where P1 and the other six cases of underage males lived together was a relatively far away from the urban area of the demolition and resettlement of the community, and the surrounding area was mostly a construction site or a factory. The residents of the neighborhood are mainly low-income groups, such as the older adults, children, and tenants and their children, who have been relocated and resettled. The poor living conditions and economic status further increase the risk of these LBC being victimized by adult MSM through monetary inducement or violent coercion. Six of P2’s seven underage sex partners were school dropouts, with the youngest having sex for the first time at the age of 13 and the oldest at the age of 16. The prevention and control of HIV among adolescents has been mainly focused on school children, but the present epidemic suggests that the prevention and control of HIV among left-behind and out-of-school children in the younger age groups should also be emphasized (20). Although there is currently a lack of cases involving intentional transmission of HIV to LBC, from the perspective of global judicial practice, acts of intentional HIV transmission have become a key focus of legal regulation in many countries due to their severe infringement on others’ rights to life and health. In recent years, a number of representative prosecution and judgment cases have emerged both domestically and internationally (23, 27–29). These cases not only reflect the negative legal evaluation of such harmful acts, but also embody the judicial logic of act characterization and liability determination under different legal systems. At present, there is a lack of research on the level of HIV infection and factors affecting vulnerable groups such as LBC, out-of-school children and children from low-income families, and the next step should be to explore the research on the prevention and control of HIV infection in these vulnerable groups.

In addition, P3, P5, P6, P7, and P8 were all friends of P1 in the same neighborhood, and all of them were introduced to P2 by P1 and had homosexual sex with P2. This again exposes the risk of HIV-infected individuals transforming their social networks into transmission networks (30). Previous studies have concluded that the risk of HIV transmission from men who have sex with men to heterosexuals is low (12, 31). In contrast, the seven underage males involved in this outbreak were all heterosexually oriented, yet all had homosexual sex with P2. This also reveals the risk of HIV infection and transmission in heterosexually oriented males who are induced by money and coerced by violence to have same-sex sex with MSM populations (32). P2 was finally sentenced to 17 years of imprisonment for the crimes of intentional injury, indecent assault on a child, and transmitting sexually transmitted infections and was sentenced to a reduction of 3 years of imprisonment due to the active explanation of the crime, which provides a reference basis for the conviction of intentional transmission of HIV in China’s judicial field. Reference basis for the conviction of intentional HIV transmission in China’s judicial field.

There are some limitations in this study, firstly, P1 and P2 failed to clarify the time of infection due to the lack of history of previous negative HIV test. Secondly, P2 had a large number of sexual partners and complex relationships within and outside Zhejiang Province. Although the CDC and public security departments conducted an in-depth investigation of P1’s and P2’s social relationships in Jiaxing, they were unable to investigate all of P2’s sexual partners in other provinces. Ultimately, the source of P2’s HIV infection could not be determined. Third, although local CDC and public security departments conducted a rigorous and systematic investigation and verification of testimonies provided by the participants enrolled in this study, inevitable recall bias arose due to the extensive time frame involved and the large cohort of individuals implicated. This bias, in turn, compromised the granularity of the transmission network characterization. In this study, when the evidence of epidemiological investigation was not fully sufficient, HIV molecular evolution analysis could still determine the transmission relationship between the investigated subjects, which once again demonstrated the important role of this biological technique in analyzing the transmission association between HIV-infected individuals (33).

In summary, underage heterosexual males are still at risk of HIV infection through men who have sex with men, and the policy and social attention to preventing and controlling the AIDS epidemic should be further strengthened for minors, especially for disadvantaged groups such as children who are out of school and those who stay behind.

## Data Availability

The datasets used and analyzed during the current study are available from the corresponding author on reasonable request.

## Footnotes

1
http://hiv-web.lanl.gov/content/index
