# Correction: A transmission relationship investigation of HIV infection through male-to-male sex among a case of left-behind children with heterosexual orientation in Zhejiang Province of China

**DOI:** 10.3389/fpubh.2026.1826213

**Published:** 2026-05-04

**Authors:** Zhigang Hou, Jiafeng Zhang, Hao Feng, Zhongwen Chen, Qin Fan, Xiaohong Pan, Guoying Zhu, Rui Ge

**Affiliations:** 1 Jiaxing Center for Disease Control and Prevention, Jiaxing, China; 2 Department of HIV/AIDS Control and Prevention, Zhejiang Provincial Center for Disease Control and Prevention, Hangzhou, Zhejiang, China; 3 Zhejiang AIDS/STD Prevention Association, Hangzhou, Zhejiang, China

**Keywords:** HIV, left-behind children, MSM, phylogenetic analysis, transmission relationship

The legend of Figure 2 was erroneously displayed as

“Bayesian phylogenetic tree of consensus sequences from the P1, P2, and local controls. (A) Bayesian phylogenetic tree for consensus gag sequences and reference sequences. The scale bar indicates 1% nucleotide sequence divergence. (B) Bayesian phylogenetic tree for consensus pol sequences and relative reference sequences. The scale bar indicates 1% nucleotide sequence divergence. (C) Bayesian phylogenetic tree for consensus env sequences and relative reference sequences. The scale bar indicates 1% nucleotide sequence divergence. Values on the branches represent the percentage of 1,000 bootstrap replicates and bootstrap values over 70% are shown in the tree. Red round: the sequence from the P1; Blue square: the sequences from the P2-1; Blue triangle: the sequences from the P2-2; Rhombic box: locally prevalent strain.”

The corrected legend of Figure 2 appears below.

“Bayesian phylogenetic tree of consensus sequences from the P1, P2, and local controls. **(A)** Bayesian phylogenetic tree for consensus *gag* sequences and reference sequences. The scale bar indicates 1% nucleotide sequence divergence. **(B)** Bayesian phylogenetic tree for consensus *pol* sequences and relative reference sequences. The scale bar indicates 1% nucleotide sequence divergence. **(C)** Bayesian phylogenetic tree for consensus *env* sequences and relative reference sequences. The scale bar indicates 1% nucleotide sequence divergence. Values on the branches represent the percentage of 1,000 bootstrap replicates and bootstrap values over 70% are shown in the tree. Red round: the sequence from the P1; Blue square: the sequences from the P2-1; Blue triangle: the sequences from the P2-2; Yellow dashed line: HIV gene sequences of the study subjects cluster together in the phylogenetic tree; Rhombic box: locally prevalent strain.”

There was a mistake in the **Acknowledgements** as published. The corrected **Acknowledgements** appears below.

“We are very grateful to the community health workers for their participation and support in the epidemiologic investigation.”

In the **Abstract**, a sentence was erroneously given as “HIV DNA was extracted, and specific gene regions were amplified, cloned, and sequenced. Phylogenetic trees were created using MEGA V6.0 to identify HIV subtypes, calculate genetic distances, and analyze genetic associations.” The correct sentence is “HIV RNA was extracted, and specific gene regions were amplified, cloned, and sequenced. Phylogenetic trees were constructed using MEGA V6.0 to identify HIV subtypes, calculate genetic distances, and analyze genetic associations.”

In the **Abstract**, the text was erroneously given as “P2 is an adult, did not communication with P1 about his HIV status and had unprotected sex with P1 before P1 tested HIV positive.” The correct text is “P2 is an adult, did not communicate with P1 about his HIV status and had unprotected sex with P1 before P1 tested HIV positive.”

In the **Abstract**, the text was erroneously given as “P2 reported seven male sexual partners in same district, P1 was one of them and all other sexual partners were HIV-negative.” The correct text is “P2 reported seven male sexual partners in the same district, P1 was one of them and all other sexual partners were HIV-negative.”

In the **Abstract**, the text was erroneously given as “Phylogenetic analysis revealed that both P1 and P2 had CRF01_AE/CRF55_01B/CRF07_BC recombinant HIV, with genetic similarities in the gag, pol, and env regions of 98.8, 99.5, and 98.8% for P1 and 99.0, 99.7, and 99.0% for P2.” The correct text is “Phylogenetic analysis revealed that both P1 and P2 had CRF01_AE/CRF55_01B/CRF07_BC recombinant HIV. The genetic similarity of the P1 sample and the two P2 samples in the gag, pol, and env gene regions was 98.8%, 99.5%, and 98.8%, and 99.0%, 99.7%, and 99.0%, respectively.”

In the **Abstract**, the text was erroneously given as “Epidemiological and phylogenetic analyses concluded that P1 contracted HIV through unprotected sex with an HIV-positive P2 who did not disclosure his HIV infection.” The correct text is “Epidemiological and phylogenetic analyses suggest that P1 may have contracted HIV through unprotected sex with an HIV-positive P2 who did not disclose his HIV infection.”

There was an error in the text of **Results**, subsection *Transmission relationship*, Paragraph 2. The text was erroneously given as “The genetic similarity of P1 and P2-1 in the gag, pol and env gene regions were 98.8, 99.5, and 98.8%, respectively; the genetic similarity of P1 and P2-2 in the gag, pol and env gene regions were 99.0, 99.7, and 99.0%, respectively. The genetic similarity of P2-1 and P2-2 in the gag, pol and env gene regions were 98.7, 99.6, and 99.9%, respectively. Env gene regions were 98.7, 99.6, and 99.9% genetically similar, respectively (Table 1).” The correct text is “The genetic similarity of P1 and P2-1 in the gag, pol, and env gene regions were 98.8%, 99.5%, and 98.8%, respectively; the genetic similarity of P1 and P2-2 in the gag, pol, and env gene regions were 99.0%, 99.7%, and 99.0%, respectively. The genetic similarity of P2-1 and P2-2 in the gag, pol, and env gene regions were 98.7%, 99.6%, and 99.9%, respectively (Table 1).”

There was an error in the text of **Results**, subsection *Transmission relationship*, Paragraph 3. The text was erroneously given as “The average within-group genetic distances between P1 and P2 had mean intra-group genetic distances of 0.003 ± 0.001 and 0.010 ± 0.001 in the pol and env gene regions, respectively, while the mean inter-group genetic distances from control sequences were 0.058 ± 0.008 and 0.130 ± 0.010, respectively (Table 2), with statistically significant differences between the two groups (*p* < 0.001).” The correct text is “The mean within-group genetic distances of P1 and P2 in the pol and env gene regions were 0.003 ± 0.001 and 0.010 ± 0.001, respectively, while the mean within-group genetic distances between them and the control sequences were 0.058 ± 0.008 and 0.130 ± 0.010, respectively (Table 2), with statistically significant differences between the two groups (*p* < 0.001).”

There was an error in the text of **Results**, subsection *Transmission relationship*, Paragraph 4. The text was erroneously given as “In the gag and env gene regions, the paraphyletic sequences of Pl and P2 were in the same evolutionary branch, bootstrap value = 99%, and the average genetic distance within clusters ≤ 0.015, and both of them were in paraphyletic phenomenon (**Figures 2A, B**).” The correct text is “Epidemiological and phylogenetic analyses highly suggest that P1 acquired HIV through unprotected sexual contact with HIV-positive P2, who had not disclosed his HIV status. In the gag and env gene regions, the paraphyletic sequences of Pl and P2 were in the same evolutionary branch, bootstrap value = 99%, and the average genetic distance within clusters ≤ 0.015, and both of them were in paraphyletic phenomenon (**Figures 2A, B**).”

There was an error in the **Discussion**, paragraph 1. The text was erroneously given as “The genetic similarity between the two specimens of P1 and P2 in the gag, pol and env gene regions was more than 98.8%, and the higher genetic similarity of the genes represented the higher genetic homology (23, 24). The average genetic distance within the groups of P1 and P2 in the pol and env gene regions was smaller than the average genetic distance between the groups of P1 and P2 with the control sequences, and the difference was statistically significant (*p* < 0.001).” The correct text is “The genetic similarity between the two specimens of P1 and P2 in the gag, pol, and env gene regions was more than 98.8%, and the higher similarity of the genes represented the higher genetic homology (23, 24). The mean genetic distance within the groups of P1 and P2 in the pol and env gene regions was smaller than the average genetic distance between the groups of P1 and P2 with the control sequences, and the difference was statistically significant (*p* < 0.001).”

The original version of this article has been updated.

