# Supplementary figures and images for: A transmission relationship investigation of HIV infection through male-to-male sex among a case of left-behind children with heterosexual orientation in Zhejiang Province of China
Source: Front Public Health. 2026 Jan 16;14:1619949. doi: 10.3389/fpubh.2026.1619949 (PMC12855501; doi:10.3389/fpubh.2026.1619949)

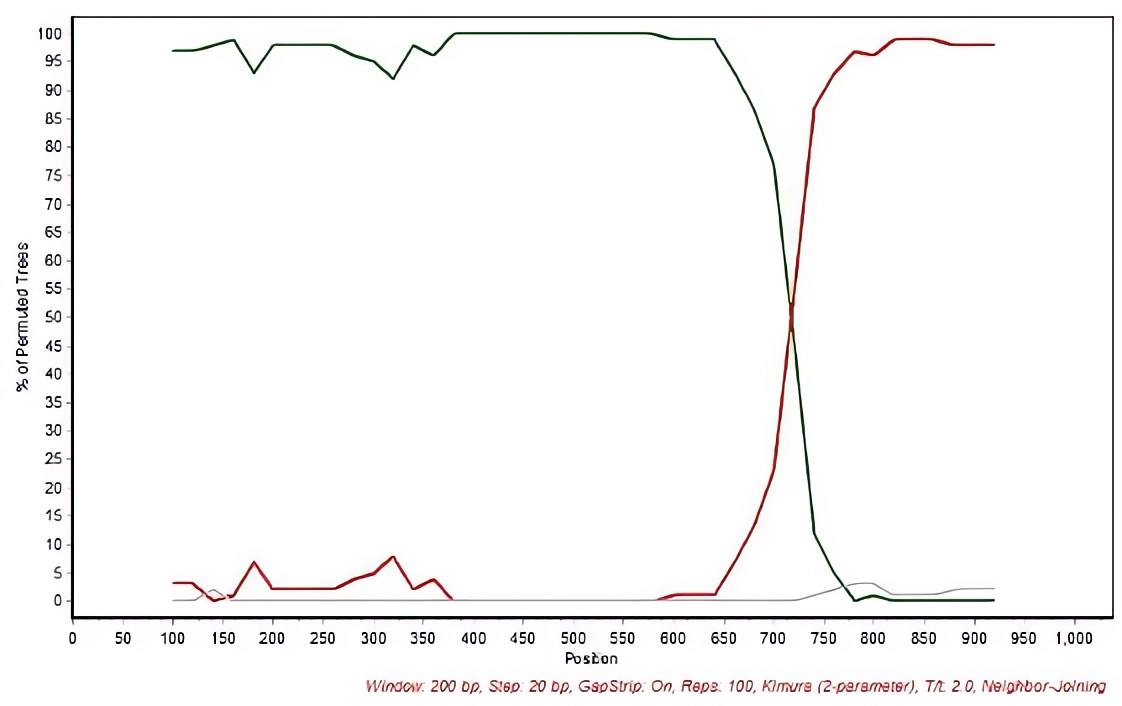


Figure S1. Structural map of HIV recombination in C1

Supplement: Supplementary file 1 [file Supplementary_file_1.docx]
