# Supplementary material for: A transmission relationship investigation of HIV infection through male-to-male sex among a case of left-behind children with heterosexual orientation in Zhejiang Province of China
Source: Front Public Health. 2026 Jan 16;14:1619949. doi: 10.3389/fpubh.2026.1619949 (PMC12855501; doi:10.3389/fpubh.2026.1619949)
Supplement: Supplementary file 2 [file Supplementary_file_2.docx]

**Methods for the homology Analysis**

Nucleic acid extraction, amplification, cloning, and phylogenetic analysis in this study were performed as follows:

**Nucleic acid extraction**

Viral RNA was extracted from 140 μl of plasma using the QIAamp Viral RNA Mini Kit (Qiagen, Valencia, CA, United States) according to the manufacturer's instructions. Following extraction, RNA was reverse-transcribed and subjected to nested polymerase chain reaction (PCR).

**Amplification of HIV-1 Gene Fragments**

**Viral RNA was reverse-transcribed and amplified** to generate fragments of gag (HXB2: 781→1861; encoding portions of p17 and p24), pol (HXB2: 2147→3462; encoding the protease and the first 299 residues of reverse transcriptase) and env (HXB2: 7002→7541, encoding the V3-V4 region), respectively.

The gag fragments were amplified using the PrimeScript^TM^ One Step RT-PCR Kit Ver.2 (Takara, Dalian, China) with primers GAG-L (5′- TCGACGCAGGACTCGGCTTGC -3′) and GAG-E2 (5′- TCCAACAGCCCTTTTTCCTAGG -3′) in 25 µl reaction volume. Cycling conditions were as follows: 50°C for 30 min, 94°C for 3 min; 94°C for 30 s, 55°C for 30 s, 72°C for 1 min 30 s, 30 cycles; 72°C for 10 min. The nested *gag* PCR was performed using the Ex Taq Kit(Takara, Dalian, China) with primers GUX (5′-AGGAGAGAGATGGGTGCGAGAGCGTC-3′) and GDX (5′- GGCTAGTTCCTCCTACTCCCTGACAT-3′) in 50 µl reaction volume. Cycling conditions were: 94°C for 3 min; 94°C for 30 s, 55°C for 30 s, 72°C for 1 min 30 s, 30 cycles; 72°C for 10 min.

The *pol* fragment was amplified with primers MAW26-07BC (5′-TGGAAATGTGGAAAAGAAGGAC-3′) and RT21-07BC (5′-CTGTATTTCAGCTATCAAGTCTTTTGATGGG-3′) using the PrimeScript™ One Step RT-PCR Kit Ver.2 with cycling conditions: 50°C for 30 min; 94°C for 2 min; 94°C for 30 s, 55°C for 30 s, 72°C for 2 min 30 s, 30 cycles;72°C for 10 min. The nested *pol* PCR was performed using the Ex Taq kit with primers PRO1-07BC (5′-CAGAGCCAACAGCCCCACCA-3′) and RT20-07BC (5′-CTGCCAATTCTAATTCTGCTTC-3′) with cycling conditions as following: 94°C for 5 min; 94°C for 30 s, 63°C for 30 s, 72°C 2 min 30 s, 30 cycles; 72°C for 10 min.

The *env* fragment was amplified with primers 44F (5′-ACAGTRCARTGYACACATGG-3′) and 35R (5′-CACTTCTCCAATTGTCCITCA-3′) using the PrimeScript™ One Step RT-PCR Kit Ver.2 with cycling conditions: 50°C for 30 min, 94°C for 3 min; 94°C for 30 s, 50°C for 30 s, 72°C for 2 min, 30 cycles; 72°C for 10 min. The nested *env* PCR was performed with primers DR7 (5′- CTGTTAAATGGYAGYCTAGC -3′) and DR8 (5′- CTCCAATTGTYCCTCATAT -3′) using the Ex Taq kit with cycling conditions as: 94°C for 2 min; 94°C for 30 s, 54°C for 30 s, 72°C 1 min, 5 cycles; 94°C for 30 s, 58.5°C for 30 s, 72°C for 1 min, 25 cycles; 72°C for 10 min.

The target PCR products were purified and sequenced by Hangzhou TsingKe Biotech Co., Ltd. (Hangzhou, China) using an ABI 3730XL DNA sequencer (Applied Biosystems, Carlsbad, CA, USA) with overlapping primers.

**TA Cloning and Sequencing**

TA cloning was performed commercially by Hangzhou TsingKe Biotech Co., Ltd. (Hangzhou, China). Briefly, PCR amplicons were purified using gel extraction kit and ligated into a TA vector following the manufacturer's protocol. The ligation mixture was transformed into competent *E. coli* DH5α cells via heat-shock method, and positive clones were selected on LB agar plates **supplemented with ampicillin**. White colonies were picked and cultured overnight in LB broth with ampicillin. Plasmid DNA was isolated and screened for correct insert size by colony PCR using M13 universal primers. For each sample, a minimum of 20 independent clones per HIV-1 gene region (gag, pol, env) were randomly selected and subjected to Sanger sequencing using the ABI 3730XL platform (Applied Biosystems, Foster City, CA, USA) with M13 forward and reverse primers.

Positive and negative controls were established through nucleic acid extraction, PCR amplification and molecular cloning. No nucleic acid cross-contamination occurred during the experiment.

**Phylogenetic Analysis**

Sequences derived from the same gene region for each sample were assembled using Sequencher v5.4.6 (Gene Codes Corporation, Ann Arbor, MI, USA). Multiple sequence alignments were performed with ClustalW implemented in BioEdit v7.2. Reference sequences, encompassing major HIV-1 subtypes and circulating recombinant forms (CRFs), were retrieved from the Los Alamos HIV Sequence Database ([https://www.hiv.lanl.gov/content/index](https://www.hiv.lanl.gov/content/index)). Genetic distances between nucleotide sequences were estimated using the Kimura 2-parameter model in MEGA v6.0. As controls, sequences from the most closely related subtype/CRF strains identified among newly diagnosed HIV-1 infections in Jiaxing City during 2018 were included.

**Phylogenetic trees were constructed via the neighbor-joining method with bootstrap resampling (1,000 replicates).** Subtype assignment was performed as follows: Viral sequences were assigned to subtypes/CRFs based on clustering with reference strains at phylogenetic nodes supported by bootstrap values >75%. Sequences not clustering with known subtypes or CRFs were classified as unique recombinant forms (URFs). Potential inter-subtype recombinations in candidate sequences were assessed using the Recombination Identification Program (RIP) v3.0.

Clusters indicative of transmission linkage were defined as monophyletic groups with bootstrap values ≥90% and mean genetic distances ≤1.5% (0.015 substitutions/site). The degree of transmission association was further assessed by evaluating sequence interspersion (i.e., interwoven branching patterns) within clusters to infer epidemiological connectivity.
